# A Real-World Study on Diagnosis and Treatment of Uterine Sarcoma in Western China

**DOI:** 10.7150/ijbs.39773

**Published:** 2020-01-01

**Authors:** Dan Li, Na Yin, Guobo Du, Shaohua Wang, Zhibo Xiao, Jinyun Chen, Wenzhi Chen

**Affiliations:** 1 Key Laboratory of Ultrasound Engineering in Medicine Co-Founded by Chongqing and the Ministry of Science and Technology, College of Biomedical Engineering, Chongqing Key Laboratory of Biomedical Engineering, Chongqing Medical University, Chongqing Collaborative Innovation Center for Minimally-invasive and Noninvasive Medicine, Chongqing 400016, China.; 2 Department of Obstetrics and Gynecology, Research Institute of Surgery, Daping Hospital, Army Medical University, Chongqing 400038, China.; 3 Department of Oncology, The Affiliated Hospital of North Sichuan Medical College, Nanchong 637000, China.; 4 Department of Pathology, The Affiliated Hospital of Southwest Medical University, Luzhou 646000, China.; 5 Department of Radiology, First Affiliated Hospital of Chongqing Medical University, Chongqing 400042, China.; 6 Ultrasound Ablation Center, First Affiliated Hospital of Chongqing Medical University, Chongqing 400042, China.; 7 Clinical Center for Tumor Therapy, The Second Affiliated Hospital of Chongqing Medical University, Chongqing 400010, China.

**Keywords:** adjuvant therapy, China, preoperative diagnosis, uterine sarcoma, survival

## Abstract

Uterine sarcomas constitute a rare heterogeneous group of gynecological malignancies with aggressive characteristics and poor prognosis. They have similar clinical features to benign leiomyomata making them difficult to reliably identify prior to hysterectomy. The preoperative prediction of uterine sarcoma remains a clinical dilemma. The current study conducted a multicentre, retrospective study to examine the accuracy of preoperative diagnosis, the consequent influence on therapy, and survival factors in patients with uterine sarcoma in Western China. Four affiliated hospitals of the medical college in Western China over a six-year period. One hundred and fourteen patients diagnosed with low-grade endometrial stromal sarcoma (LG-ESS), high-grade endometrial stromal sarcoma (HG-ESS), undifferentiated uterine sarcoma (UUS), leiomyosarcoma (LMS), or adenosarcoma (AS) were analyzed. The median age at diagnosis was 47 years. Eighty (70.2%) patients were premenopausal and 34 (29.8%) post-menopausal. The most common pathological type was LG-ESS (43.9%). The diagnostic sensitivity of ultrasound for uterine malignant tumors was 11.0%, much lower than MRI (35.3%) and CT (63.0%). Unlike MRI, most of the patients who underwent CT (88.2%) examination were at the advanced stage. Forty-seven (41.2%) patients with uterine sarcoma were diagnosed with uterine malignant tumor before operation. Thirty-two (47.8%) patients who were misdiagnosed before operation needed reoperation and five patients (4.6%) diagnosed after radical surgery developed distant metastasis simultaneously. The recommended treatment of 87.0% of the patients with uterine sarcoma was total hysterectomy and bilateral salpingooophorectomy, and 53.7% of patients received adjuvant chemotherapy after operation. Pelvic lymph node status were clarified in 47 patients (43.5%), which were higher in HG-ESS and UUS groups, and lower in LMS group (*P* = 0.013). In univariate analysis, we found a significant association between tumour histological types, tumour stage, menstrual status, elevated preoperative neutrophil/lymphocyte ratio and overall survival. In multivariate analysis, we only observed a significant association between tumour histological types and tumour stage and overall survival.

## Introduction

Uterine sarcomas are rare and aggressive tumors, accounting for approximately 1% of all female genital tract malignancies and 3-7% of all uterine malignances 1. Their rarity and histopathological diversity have contributed to the lack of a consensus on the prognostic factors and optimal treatment 2-4. According to the 2003 World Health Organization (WHO) classification, uterine sarcomas are classified into pure and heterologous sarcomas. The pure mesenchymal tumors are further classified into leiomyosarcoma (LMS), endometrial stromal sarcoma (ESS), and undifferentiated uterine sarcoma (UUS). The heterologous tumors include carcinosarcoma (CS) and adenosarcoma (AS) 5. Subsequently, carcinosarcoma has been reclassified as a dedifferentiated or metaplastic form of endometrial carcinoma 6. Endometrial sarcomas are further classified into the following two main categories: (1) low-grade endometrial stromal sarcoma (LG-ESS); and (2) high-grade endometrial stromal sarcoma (HG-ESS) by the 2014 WHO classification 7. The prognosis of uterine sarcoma considerably varies according to pathologic type, tumour stage; age and tumour size 2-4. Surgery correctly performed is imperative and is the most important prognostic factor for most patients. Patients with preoperative suspected uterine sarcoma should be referred to specialist centers where appropriate surgery can be performed. The lack of specific symptoms, signs, or diagnostic techniques for preoperative differentiation from uterine fibroids results in unexpected uterine sarcomas after surgery 8, 9. If a uterine sarcoma is mistakenly diagnosed as a uterine fibroid and is morcellated via laparoscopy, then serious consequences may arise 10. Due to the lack of standard treatment for uterine sarcomas, clinicians not only face unexpected uterine sarcomas after surgery, but also consider what is the optimal surgical procedure and adjuvant therapy for these patients. Due to the incomplete staging information, unnecessary surgical procedure is also inevitable. The present study aimed to examine the accuracy of preoperative diagnosis, the consequent influence on therapy, and survival factors in patients with uterine sarcoma in Western China.

## Materials and Methods

### Study subjects

A retrospective chart review was performed at four affiliated hospitals of medical college in Western China: the first affiliated hospital of Chongqing Medical University in chongqing and the Daping Hospital affiliated to the Army Medical University in chongqing, the affiliated Hospital of North Sichuan Medical College in Nanchong, Sichuan Province, and the affiliated Hospital of Southwest Medical University in Luzhou, Sichuan Province. As this was a retrospective analysis, the ethics committee of these hospitals waived the need for written informed consent from participants. Verbal consent was obtained from each surviving patient and the family of patients who were deceased by the phone call follow-up.

Pathology databases at each institution were searched to identify patients with any type of uterine sarcoma diagnosed between January 1, 2010 and January 1, 2016. The pathology reports were reviewed, and patients diagnosed with LG-ESS, HG-ESS, UUS, LMS, and AS were included. All other sarcomas and patients with recurrent sarcomas were excluded. All patients were newly diagnosed and treated with complete medical history.

Study parameters included age at the time of surgery, menstrual status, clinical findings, blood test results, imaging examinations results, preoperative pathological diagnosis, frozen section diagnosis, postoperative pathological diagnosis, details of the surgical procedures and adjuvant therapy. The primary surgical procedure was defined as the first major surgery (hysterectomy or myomectomy) when sarcoma was either diagnosed or when an attempt at cure was made. Endometrial sampling procedures (hysteroscopy /dilatation and curettage) were noted but not classified as the primary surgical procedure. Data on history of previous cancer diagnoses and history of radiation treatment were extracted.

Patients were followed for overall survival (OS) outcomes. OS was measured from the initiation of histopathological confirmation to death of any cause. Patients without an event or lost to follow-up were censored at the time of the last follow-up visit. The cut-off point for the survival study was October 1, 2018.

### Diagnosis and staging

The diagnoses of all patients were confirmed and the histological types were ascertained by retrieving previous corresponding histological slides. Histopathological classification of uterine sarcomas was according to the 2014 WHO classification system. Patients were restaged using the 2009 International Federation of Gynecology and Obstetrics (FIGO) staging classification.

### Statistical analysis

Statistical analysis was conducted using SPSS for Windows (version 21.0, SPSS). Dichotomous variables were compared using Fisher's exact test or chi-squared test. Continuous variables were compared using the *t-*test or nonparametric test. The optimal cutoff values for the neutrophil /lymphocyte (N/L) ratio were calculated by applying receiver operating curve (ROC) analysis. In the multivariate Cox-regression analysis, the model was adjusted for prognostic factors significantly associated with OS in univariate analysis. Hazard ratio (HR) estimated from the Cox analyses were reported as relative risks with corresponding 95% confidence interval (CI). The association between clinical and N/L ratio with OS was analysed using Kaplan-Meier curves and compared by the log- rank test. For all statistical tests, *P* < 0.05 was considered significant.

## Results

### Patients' characteristics

A total of 114 uterine sarcoma cases were confirmed for the histological diagnosis and retrieved for this retrospective analysis. Prior histories of cancer were documented in two patients (1.8%). One patient was previously diagnosed with breast cancer, and one had cervical cancer which had previously undergone pelvic radiation treatment. The median age at diagnosis was 47 years (range 20 to 79 years). Eighty (70.2%) patients were premenopausal and 34 (29.8%) post-menopausal. The most common pathological type was LG-ESS (43.9%), followed by LMS (29.8%), and AS (7.0%) was the least common pathological type. Compared with the LG-ESS and HG-ESS groups, the UUS group had the oldest average patient age, of which most were postmenopausal patients. The average tumor size was the largest in LMS group and was statistically significant compared with the LG-ESS group and AS group (*P* = 0.005).

The diagnostic sensitivity of ultrasound for uterine malignant tumors was 11.0%, much lower than MRI (35.3%) and CT (63.0%). Unlike MRI, most of the patients who underwent CT (88.2%) examination were at the advanced stage. Ultrasound had the highest diagnostic sensitivity in UUS group (33.3%), but the difference was not statistically significant.The value of preoperative serum tumor marker CA125 and LDH for the diagnosis of uterine sarcomas is limited, and the preoperative CA125 abnormality and LDH abnormality were found in 24 and 17 of 70 cases, respectively. Forty-two (36.8%) patients were diagnosed with an invasive tumor by preoperative pathology. The majority of patients were preoperatively diagnosed by endometrial sampling (85.7%), and a few by vaginal neoplasm biopsy (14.3%). Endometrium sampling was performed with a blinded fractional curettage (58.3%) or guided by hysteroscopy (41.7%). A total of 48 patients in this study underwent endometrial sampling, and 75% (36 of 48) were diagnosed preoperatively with a uterine malignancy. There was no statistical difference in diagnostic sensitivity between the two endometrium sampling methods (*P* = 0.051). The majority of women with uterine sarcoma presented stage I disease (71.9%). Additional demographics for the study population are shown in **Table 1**.

### Preoperative diagnosis and surgical arrangement

Forty-seven (41.2%) patients with uterine sarcoma were diagnosed with uterine malignant tumors before operation, five (4.4%) patients were diagnosed by typical imaging findings, and 41 patients underwent a surgical procedure tailored for uterine malignant tumor, with 87.8% following the operation principles for uterine sarcoma. Approximately 79.4% of the LMS cases were misdiagnosed preoperatively, and no patient in the UUS group misdiagnosed before operation. Of the 67 patients (58.8%) who were diagnosed with non-malignant tumors before operation, 22 (32.8%) underwent intraoperative frozen section examination and were highly suggestive of a malignant tumor, including the cases that needed further confirmation. According to the results obtained from frozen section examination, 17 cases (25.4%) had corrected the surgical procedure and changed to the extended range of malignant tumor resection. There were still 32 (47.8%) patients needing reoperation because of non-routine intraoperative frozen section examination, negative freezing results or deferred diagnosis. However, only 18 patients (26.9%) completed. The rest of the 14 (20.9%) patients who were misdiagnosed before operation were not recommended to undergo reoperation because the initial treatments were total abdominal hysterectomy with or without bilateral salpingo-oophorectomy. Surgical arrangements for preoperatively misdiagnosed patients are shown in **Table 2.**

### Comprehensive treatment and survival results

**Table 3** summarizes the treatment for uterine sarcoma patients and shows that, of the 108 patients who underwent surgery, 73.1% underwent abdominal hysterectomy surgeries, 22.1% underwent laparoscopic hysterectomy surgeries, and only 4.6% were diagnosed with uterine sarcoma at the time of open or hysteroscopic myomectomy. Approximately 87.0% patients underwent bilateral salpingo- oophorectomy surgeries. Pelvic lymph node status were clarified in 47 patients (43.5%), which were higher in HG-ESS and UUS groups, and lower in LMS group (*P* = 0.013). Only one patient (0.9%) with LMS had morcellation during their primary surgery. Five patients (4.6%) diagnosed with uterine sarcoma after radical surgery simultaneously developed distant metastasis. Adjuvant chemotherapy were given to 53.7% patients, the vast majority of patients in HG-ESS and UUS groups. Most commonly used chemotherapy combinations were doxorubicin/ ifosfamide and doxorubicin/ dacarbazine, with 4 to 6 cycles. Adjuvant radiotherapies were performed in 17.6% patients. Hormonal therapies were only used to prevent recurrence of LG-ESS (66.7%) in present study.

## Discussion

This retrospective review conducted at four affiliated hospitals of medical college in Western China over a six-year period provides insights into the preoperative diagnosis, treatment and survival of uterine sarcoma. The most frequent type of uterine sarcoma was LG-ESS, which was found in about 43.9% cases, followed by LMS (29.8%) and HG-ESS (11.4%), which was in line with earlier studies on the clinical characteristics of uterine sarcoma in China 11-13. As ESS was the most common pathological subtype, 76.0% of which in premenopausal patients, and the average age of uterine sarcoma patients in the current study was lower than other retrospective studies 8-9, 11. The UUS group had the oldest average patient age, and most of which in postmenopausal patients, similar to the findings of a previous study 14.

**Figure 1:**
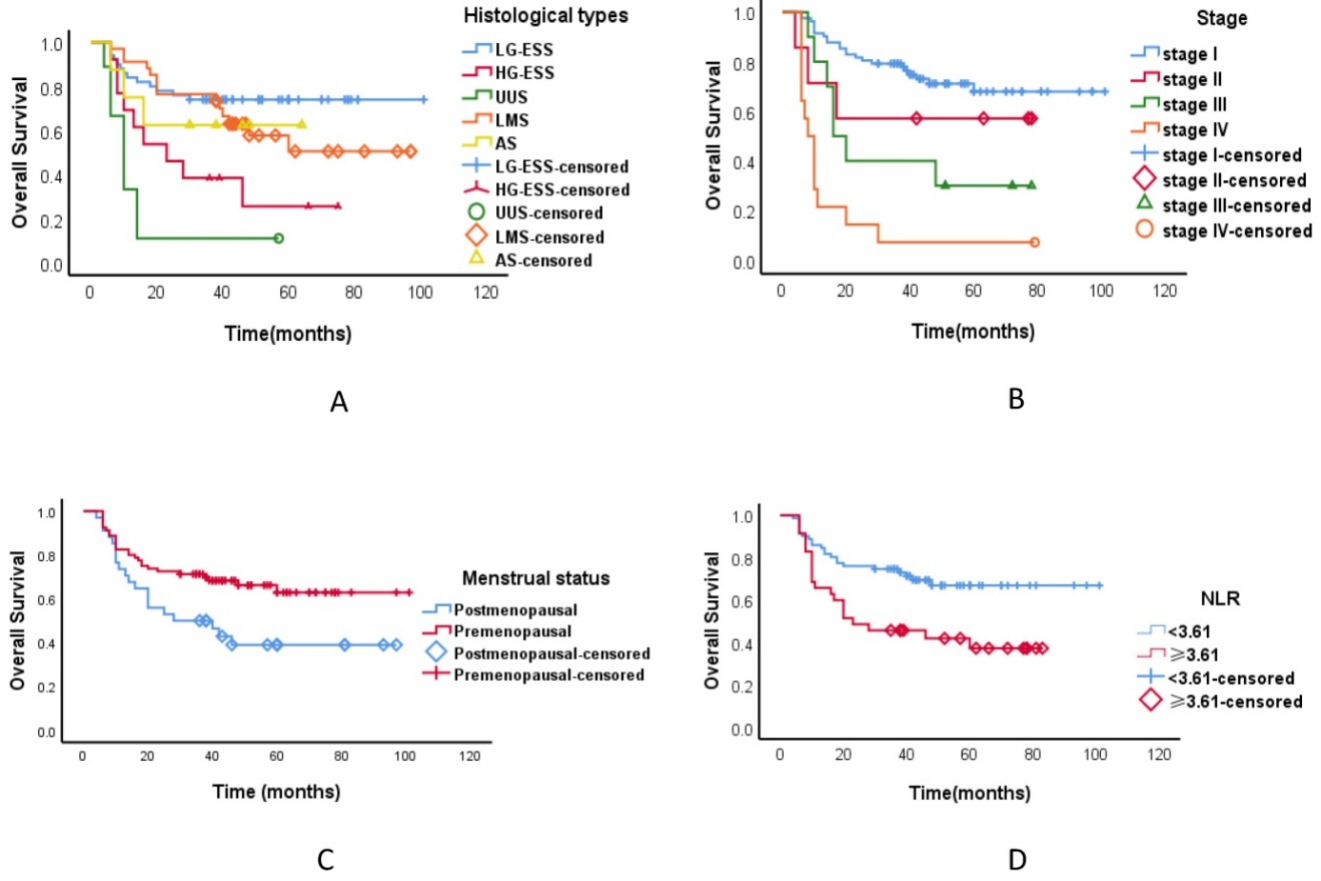
** Kaplan-Meier estimates of overall survival curves of patients with uterine sarcoma.** A.The overall survival of different pathological types; B.The overall survival of different stages; C.The overall survival in different menstrual states; D. The neutrophil/lymphocyte ratio and overall survival. Abbreviations: LG-ESS: low-grade endometrial stromal sarcoma; HG-ESS: high-grade endometrial stromal sarcoma; UUS: undifferentiated uterine sarcoma; LMS: leiomyosarcoma; AS: adenosarcoma; NLR: neutrophil/lymphocyte ratio.

As of October 2018, the median duration of follow-up was 37 months, ranging from 6 to 100 months. The follow-up rate was 90.6%. Applying ROC analysis the optimal cutoff value for N/L ratio was 3.61 for OS. In univariate analysis, we found a significant association between tumour histological types, tumour stage, menstrual status, elevated preoperative N/L ratio and OS (**Tables 4**, **Figure 1**). In multivariate analysis, we observed a significant association between tumour histological types and tumour stage and OS (**Tables 4**). The three-year OS was 60.2% for all the patients with uterine sarcoma, 71.1% for LG-ESS, 71.4% for LMS, 57.1% for AS, 27.3% for HG-ESS, and 11.1% for UUS. The three-year OS of different stages of uterine sarcoma were as follows: stage I 75.4%, stage II 57.1%, stage III 33.3%, and stage IV 7.1% (*P* < 0.001).

Preoperative diagnosis of uterine sarcoma is challenging. There are no effective preoperative diagnostic modalities for uterine sarcoma. Several reports suggested that computed tomography (CT), magnetic resonance imaging (MRI), and serum lactate dehydrogenase (LDH) may be helpful in the diagnosis of uterine sarcoma 8, 15. However, given the cost-effectiveness, those diagnostic tools cannot be applied to all patients with uterine masses. Ultrasound is an initial diagnostic tool for patients with abnormal vaginal bleeding or pelvic pain, as well as for the evaluation of uterine disease 16. In the present study, the diagnostic sensitivity of ultrasound for uterine malignant tumors was 11.0%, which was much lower than MRI (35.3%) and CT (63.0%). However, 88.2% patients diagnosed by CT examination were in the advanced stage. Ultrasonography was highly sensitive in the diagnosis of UUS, and only 22.2% of patients were misdiagnosed as uterine fibroids before operation. For the high-grade or poorly differentiated endometrial stromal sarcoma, routine imaging diagnosis all showed a higher accuracy compared with the LG-ESS group. For LMS, which arises within the uterine smooth muscle, routine imaging diagnosis was difficult, unless in the advanced stages. Serum CA125 and LDH for the diagnosis of uterine sarcomas are limited in present study, and the preoperative CA125 and LDH abnormalities were detected in 24 and 17 of 70 cases, respectively.

The most reliable preoperative diagnostic method has been found to be tumor biopsy 17. As previously noted, of the 57 patients who underwent tumor biopsy, 42 (73.7%) were positive for an invasive tumor. Many studies have examined various preoperative diagnostic tools. Wais et al. 9 identified 302 patients with uterine sarcoma, and 114 (51%) underwent preoperative endometrial sampling. Pathology suggested uterine sarcoma in 65% of cases. They presumed that the indication for the sampling was likely due to abnormal uterine bleeding, especially in postmenopausal women. Of the 74 patients (24.5% of this cohort) diagnosed during endometrial sampling, the median age was 58 (range 33 to 89 years), and 51 (69.3%) were postmenopausal. Similarly, Bansal et al. 18 identified 142 patients with uterine sarcoma, and 72 (51%) underwent preoperative endometrial sampling. Pathology suggested an invasive tumor in 86% of cases and predicted the correct histologic diagnosis in 64% of patients. In the latter study, the pathological types of uterine sarcoma included carcinosarcoma, however, for the Wais's study, only LMS and ESS subtypes. In terms of preoperative diagnosis of uterine sarcoma, the CS group showed higher positive cytological results from the endometrial cavity. Cytological abnormalities were rarely detected in the LMS and ESS groups 8. Our study confirms these findings and found that 75% (36/48) of the patients who underwent preoperative endometrial sampling were diagnosed preoperatively with a uterine malignancy. We were unable to ascertain the indication for the sampling, and presume it was likely due to the hysteroscopic endometrial biopsy that we had a higher predictive value in diagnosing LMS and LG-ESS.

By performing a tumor biopsy before surgery, we can diagnose whether the uterine mass is benign or malignant. Thus, we can avoid reoperation or unneeded surgery in cases with distant metastasis or only perform limited procedures such as fertility-sparing surgery, laparoscopic surgery, or transvaginal surgery. In our study, five patients (4.6%) diagnosed with uterine sarcoma after radical surgery were found distant metastasis simultaneously, and one patient (0.9%) with LMS had morcellation during their primary surgery. Frozen section examination can assist the surgeon to tailor the extent of surgery necessary for the individual patient. While the accuracy of frozen section is well documented, errors, or deferred diagnosis may be impossible to avoid due to various factors 19. In our study, of the 25 patients who underwent intraoperative frozen section examination, 22 (88.0%) were highly suggestive of a malignant tumor, including the cases that needed further confirmation. According to the results obtained from frozen section examination, 17 cases (77.3%) had corrected the surgical procedure and changed to the extended range of malignant tumor resection. Patients' and physicians' understanding of the risks is the foundation of medical decision-making. Whether reoperation or not is affected by many factors. Fourteen (20.9%) patients who were misdiagnosed before operation were not recommended to undergo reoperation by the doctor after surgery because the initial treatments were total abdominal hysterectomy with or without bilateral salpingo-oophorectomy.

Surgery correctly performed is imperative and is the most important prognostic factor for uterine sarcoma 2-3. The standard treatment for LMS, ESS, UUS, and AS is total hysterectomy. In postmenopausal women, bilateral salpingo-oophorectomy (BSO) is also recommended. BSO has traditionally been recommended, even in premenopausal women with stage I ESS disease 2. However, several studies failed to show that BSO affects time for recurrence or OS in stage I disease. Regarding the adverse effects of early surgical menopause, preservation of the ovarian function may be an option for premenopausal women with stage I disease 20. Preservation of ovarian tissues in women with stage I LMS does not increase the risk of recurrence indicating that preservation of the ovaries in premenopausal women may be possible unless these tissues show macroscopic involvement 21. Resection of lymph nodes is also controversial. The risk of lymph node metastases has been reported to be very low; thus, routine pelvic lymphadenectomy was not routinely advised unless the lymph nodes were clinically suspected for metastasis. However, these lymph node tumors are often diagnosed postoperatively 22-23. The value of post-operative radiotherapy, if any, was to reduce local recurrence and improve local disease, but it had no effect on the overall survival 4. The role of adjuvant chemotherapy is even more poorly defined than radiation therapy for patients with localized disease but has been considered because of high risk for distant relapse 2. Due to the lack of standard treatment for uterine sarcomas, clinicians not only face unexpected uterine sarcomas after surgery, but are also confused in terms of selecting the optimal surgical procedure and adjuvant therapy. In the present study, the recommended treatment for 87.0% of the patients with uterine sarcoma was total hysterectomy and bilateral salpingooophorectomy, and 53.7% of patients received adjuvant chemotherapy after operation. In this study, the preservation of ovarian function remains a dilemma, particularly the younger patients affected by early stage ESS. In addition, for most stage I patients, postoperative adjuvant chemotherapy can be avoided, especially the patients in LG-ESS group.

Consistent with other literature reports 2, 4, our prognosis was relatively good for LG-ESS, but poor for the other types. Poor survival was also associated with advanced disease stage. Recent data indicate that inflammatory cells that accumulate around neoplasms have a crucial role in tumour progression and its prognosis 24.An elevated preoperative N/L ratio predicts poor clinical outcome in soft-tissue sarcoma patients and may serve as a cost-effective and broadly available independent prognostic biomarker 25. In uterine sarcomas, the N/L ratio was shown to be more useful than serum CA 125 for the preoperative differentiation of uterine sarcomas from benign tumours 26. In univariate analysis, we found a significant association between tumour histological types, tumour stage, menstrual status, elevated preoperative N/L ratio and OS. In multivariate analysis, we only observed a significant association between tumour histological types and tumour stage and OS. However, this study included only 114 uterine sarcoma patients, had a relative short follow-up period and analysed only OS, which might be influenced by other non-tumour related factors, as the main clinical end point.

Further studies should be encouraged to improve the preoperative diagnosis methods of uterine sarcoma and understand the optimal surgical procedure for patients with uterine sarcoma. Also, prospective randomized clinical studies should be performed to evaluate the prognostic influencing factor and the value of adjuvant treatments for patients with uterine sarcoma.

## Figures and Tables

**Table 1:** The basic characteristics for the patients of uterine sarcoma

		histological types					
Basic clinical data		Total	LG-ESS	HG-ESS	UUS	LMS	AS
**No. of patients(n,%)**		114	50(43.9)	13(11.4)	9(7.9)	34(29.8)	8(7.0)
**Age(years)**							
	Median	47	46	46	58	46	48
	Range	20-79	20-68	20-63	34-66	26-71	29-79
**Tumor size(cm)**							
	Median	7.0	5.6	7.8	7.5	8.6	5.0
	Range	1.0-27.0	1.0-14.5	1.1-20.3	2.5-15.0	2.0-23.5	2.8-27.0
**menstrual status**							
	premenopausal	80(70.2)	38(76.0)	9(69.2)	2(22.2)	26(76.5)	5(62.5)
	postmenopausal	34(29.8)	12(24.0)	4(30.8)	7(77.8)	8(23.5)	3(37.5)
**US**							
	Benign tumors	56/100(56.0)	27/44(61.4)	5/12(41.7)	2/9(22.2)	19/28(67.9)	3/7(42.9)
	Malignancy suspected^*^	33/100(33.0)	14/44(31.8)	5/12(41.7)	4/9(44.4)	7/28(25.0)	3/7(42.9)
	Malignant tumors	11/100(11.0)	3/44(6.8)	2/12(16.7)	3/9(33.3)	2/28(7.1)	1/7(14.3)
**MRI**							
	Benign tumors	16/34(47.1)	8/15(53.3)	1/5(20.0)	1/3(33.3)	6/10(60.0)	0/1(0)
	Malignancy suspected	6/34(17.6)	3/15(20.0)	0/5(0)	0/3(0)	3/10(30.0)	0/1(0)
	Malignant tumors	12/34(35.3)	4/15(26.7)	4/5(80.0)	2/3(66.7)	1/10(10.0)	1/1(100)
**CT**							
	Benign tumors	5/27(18.5)	3/8(37.5)	0/5(0)	0/1(0)	2/9(22.2)	0/4(0)
	Malignancy suspected	5/27(18.5)	3/8(37.5)	1/5(20.0)	0/1(0)	1/9(11.1)	0/4(0)
	Malignant tumors	17/27(63.0)	2/8(25.0)	4/5(80.0)	1/1(100)	6/9(66.7)	4/4(100)
**Tumor marker**							
	CA125 abnormality	24/70(34.3)	11/30(36.7)	3/10(30.0)	1/6(16.7)	8/19(42.1)	1/5(20.0)
	LDH abnormality	17/70(24.3)	5/25(20.0)	2/9(22.2)	1/6(16.7)	8/22(36.4)	1/8(12.5)
**Preoperative pathological diagnosis**							
	Fractional curettage	18/28(64.3)	10/14(71.4)	2/5(40.0)	3/3(100)	1/3(33.3)	2/3(66.7)
	Biopsy under hysteroscopy	18/20(90.0)	9/9(100)	1/1(100)	4/4(100)	4/5(80.0)	0/1(0)
	Vaginal neoplasm biopsy	6/9(66.7)	2/3(66.7)	1/1(100)	0/1(0)	1/2(50.0)	2/2(100)
**FIGO stage^¶^**							
	I	82(71.9)	35(70.0)	8(61.5)	7(77.8)	27(79.4)	5(62.5)
	II	7(6.1)	3(6.0)	1(7.7)	1(11.1)	2(5.9)	0
	III	9(7.9)	4(8.0)	1(7.7)	1(11.1)	2(5.9)	1(12.5)
	IV	15(13.2)	7(14.0)	3(23.1)	0	3(8.8)	2(25.0)

LG-ESS:low-grade endometrial stromal sarcoma; HG-ESS: high-grade endometrial stromal sarcoma; UUS: undifferentiated uterine sarcoma; LMS: leiomyosarcoma; AS: adenosarcoma; ^*^ Malignancy suspected can be understood as an ambiguous conclusion; ^¶^ One patient at LG-ESS group refused further therapy after diagnosed during endometrial sampling, and had no staging.

**Table 2:** Surgical arrangements for preoperatively misdiagnosed uterine sarcoma patients

	histological types					
Surgical arrangement	Total	LG-ESS	HG-ESS	UUS	LMS	AS
No. of patients(n,%)	67(58.8)	29(58.0)	7(53.8)	0	27(79.4)	4(50.0)
**Frozen section diagnosis**	22(32.8)	10(34.5)	5(71.4)	0	5(18.5)	2(50.0)
Tailored accordingly	17(77.3)	6(60.0)	5(100)	0	4(80.0)	2(100)
Reoperation	5(22.7)	4(40.0)	0	0	1(20.0)	0
**Postoperative diagnosis**	45(67.2)	19(65.5)	2(28.6)	0	22(81.5)	2(50.0)
Reoperation recommend	27(60.0)	11(57.9)	2(100)	0	12(54.5)	2(100)
Reoperation completed^※^	13(48.1)	5(45.5)	2(100)	0	6(50.0)	0

^※^ Some patients refuse reoperation; LG-ESS: low-grade endometrial stromal sarcoma; HG-ESS: high-grade endometrial stromal sarcoma; UUS: undifferentiated uterine sarcoma; LMS: leiomyosarcoma; AS: adenosarcoma.

**Table 3:** Treatment for the patients of uterine sarcoma

	histological types					
**Treatment**	**Total**	**LG-ESS**	**HG-ESS**	**UUS**	**LMS**	**AS**
No. of patients(n/%)	114	50(43.9)	13(11.4)	9(7.9)	34(29.8)	8(7.0)
**Surgical procedures**	108(94.7)	45(90.0)	13(100)	9(100)	34(100)	7(87.5)
Myomectomy^▼^	5(4.6)	2(4.4)	0	0	3(8.8)	0
AH	79(73.1)	29(64.4)	10(76.9)	9(100)	25(73.5)	6(85.7)
LH	24(22.2)	14(31.1)	3(23.1)	0	6(17.6)	1(14.3)
BSO	94(87.0)	41(91.1)	11(84.6)	9(100)	29(85.3)	4(57.1)
PLND	47(43.5)	18(40.0)	8(61.5)	8(88.9)	10(29.4)	3(42.9)
OE	20(18.5)	7(15.6)	4(30.8)	3(33.3)	5(14.7)	1(14.3)
**Adjuvant treatment**						
chemotherapy	58(53.7)	20(44.4)	11(84.6)	7(77.8)	17(50.0)	3(42.9)
radiotherapy	19(17.6)	9(20.0)	3(23.1)	0	6(17.6)	1(14.3)
hormone therapy	30(27.8)	30(66.7)	0	0	0	0
**Palliative chemotherapy or hormone therapy**	4(3.5)	4(8.0)	0	0	0	0
**Refuse treatment**	2(1.8)	1(2.0)	0	0	0	1(12.5)

^▼^One patient in the LMS group was hysteroscopic myomectomy, and the rest of patients were open myomectomy; LG-ESS: low-grade endometrial stromal sarcoma; HG-ESS: high-grade endometrial stromal sarcoma; UUS: undifferentiated uterine sarcoma; LMS: leiomyosarcoma; AS: adenosarcoma; AH= abdominal hysterectomy; LH=laparoscopic hysterectomy; BSO=bilateral salping-oophorectomy; PLND=pelvic lymphadenectomy; OE=omentectomy.

**Table 4:** Cox risk model analysis of prognostic influencing factor for uterine sarcoma

Variable	Univariate analysis		Multivariate analysis	
	HR(95%CI)	P-value	HR(95%CI)	P-value
**Histological types**		0.001		<0.001
LG-ESS	1(referent)		1(referent)	
HG-ESS	3.626(1.543-8.518)	0.003	5.969(2.359-15.103)	<0.001
UUS	8.019(3.238-19.862)	<0.001	18.356(6.043-55.759)	<0.001
LMS	1.542(0.724-3.284)	0.261	2.294(1.011-5.203)	0.047
AS	1.707(0.486-5.999)	0.404	2.240(0.479-10.472)	0.305
**FIGO stage**		<0.001		<0.001
Ⅰ	1(referent)		1(referent)	
Ⅱ	1.876(0.563-6.258)	0.306	3.841(1.097-13.442)	0.035
Ⅲ	3.170(1.356-7.409)	0.008	3.573(1.468-8.695)	0.005
Ⅳ	8.676(4.276-17.604)	<0.001	14.451(6.359-32.841)	<0.001
**Age(years)**		0.512		
<50	1(referent)			
≥50	1.220(0.673-2.213)			
**Menstrual status**		0.021		
premenopausal	1(referent)			
postmenopausal	1.974(1.106-3.522)			
**Tumor size(cm)**		0.316		
≤5cm	1(referent)			
>5cm	1.400(0.725-2.705)			
**NLR**		0.008		
<3.61	1(referent)			
≥3.61	2.265(1.243-4.218)			

LG-ESS: low-grade endometrial stromal sarcoma; HG-ESS: high-grade endometrial stromal sarcoma; UUS: undifferentiated uterine sarcoma; LMS: leiomyosarcoma; AS: adenosarcoma; HR: hazard ratio; CI: confidence interval; NLR: neutrophil/lymphocyte ratio.
